# Coarse-Graining Approaches in Univariate Multiscale Sample and Dispersion Entropy

**DOI:** 10.3390/e20020138

**Published:** 2018-02-22

**Authors:** Hamed Azami, Javier Escudero

**Affiliations:** School of Engineering, Institute for Digital Communications, The University of Edinburgh, Edinburgh EH9 3FB, UK

**Keywords:** complexity, multiscale dispersion and sample entropy, refined composite technique, intrinsic mode dispersion and sample entropy, moving average, Butterworth filter, empirical mode decomposition, downsampling

## Abstract

The evaluation of complexity in univariate signals has attracted considerable attention in recent years. This is often done using the framework of Multiscale Entropy, which entails two basic steps: coarse-graining to consider multiple temporal scales, and evaluation of irregularity for each of those scales with entropy estimators. Recent developments in the field have proposed modifications to this approach to facilitate the analysis of short-time series. However, the role of the downsampling in the classical coarse-graining process and its relationships with alternative filtering techniques has not been systematically explored yet. Here, we assess the impact of coarse-graining in multiscale entropy estimations based on both Sample Entropy and Dispersion Entropy. We compare the classical moving average approach with low-pass Butterworth filtering, both with and without downsampling, and empirical mode decomposition in Intrinsic Multiscale Entropy, in selected synthetic data and two real physiological datasets. The results show that when the sampling frequency is low or high, downsampling respectively decreases or increases the entropy values. Our results suggest that, when dealing with long signals and relatively low levels of noise, the refine composite method makes little difference in the quality of the entropy estimation at the expense of considerable additional computational cost. It is also found that downsampling within the coarse-graining procedure may not be required to quantify the complexity of signals, especially for short ones. Overall, we expect these results to contribute to the ongoing discussion about the development of stable, fast and robust-to-noise multiscale entropy techniques suited for either short or long recordings.

## 1. Introduction

A system is complex when it entails a number of components intricately entwined altogether (e.g., the subway network of the New York City) [1]. Following Costa’s framework [2,3], the complexity in univariate signals denotes “meaningful structural richness”, which may be in contrast with regularity measures defined from entropy metrics such as sample entropy (SampEn), permutation entropy, (PerEn), and dispersion entropy (DispEn) [3,4,5,6]. In fact, these entropy techniques assess repetitive patterns and return maximum values for completely random processes [3,5,7]. However, a completely ordered signal with a small entropy value or a completely disordered signal with maximum entropy value is the least complex [3,5,8]. For instance, white noise is more irregular than 1/f noise (pink noise), although the latter is more complex because 1/f noise contains long-range correlations and its 1/f decay produces a fractal structure in time [3,5,8].

From the perspective of physiology, some diseased individuals’ recordings, when compared with those for healthy subjects, are associated with the emergence of more regular behavior, thus leading to lower entropy values [3,9]. In contrast, certain pathologies, such as cardiac arrhythmias, are associated with highly erratic fluctuations with statistical characteristics resembling uncorrelated noise. The entropy values of these noisy signals are higher than those of healthy individuals, even though the healthy individuals’ time series show more physiologically complex adaptive behavior [3,10].

In brief, the concept of complexity for univariate physiological signals builds on the following three hypotheses [3,5]:

- The complexity of a biological or physiological time series indicates its ability to adapt and function in an ever-changing environment.
- A biological time series requires operating across multiple temporal and spatial scales and so its complexity is similarly multiscaled and hierarchical.
- A wide class of disease states, in addition to ageing, which decrease the adaptive capacity of the individual, appear to degrade the information carried by output variables.

Therefore, the multiscale-based methods focus on quantifying the information expressed by the physiological dynamics over multiple temporal scales.

To provide a unified framework for the evaluation of impact of diseases in physiological signals, multiscale SampEn (MSE) [3] was proposed to quantify the complexity of signals over multiple temporal scales. The MSE algorithm includes two main steps: (1) coarse-graining technique—i.e., combination of moving average (MA) filter and downsampling (DS) process—; and (2) calculation of SampEn of the coarse-grained signals at each scale factor τ [3]. A low-pass Butterworth (BW) filter was used as an alternative to MA to limit aliasing and avoid ripples [11]. To differentiate it from the original MSE, we call this method MSE_BW_ herein.

Since their introduction, MSE and MSE_BW_ have been widely used to characterize physiological and non-physiological signals [12]. However, they have several main shortcomings [12,13,14]. First, the coarse-graining process causes the length of a signal to be shortened by the scale factor τ as a consequence of the downsampling in the process. Therefore, when the scale factor increases, the number of samples in the coarse-grained sequence decreases considerably [14]. This may yield an unstable estimation of entropy. Second, SampEn is either undefined or unreliable for short coarse-grained time series [13,14].

To alleviate the first problem of MSE, intrinsic mode SampEn (InMSE) [15] and refined composite MSE (RCMSE) [14] were developed [15]. The coarse-graining technique is substituted by an approach based on empirical mode decomposition (EMD) in InMSE. The length of coarse-grained series obtained by InMSE is equal to that of the original signal, leading to more stable entropy values. Nevertheless, EMD-based approaches have certain limitations such as sensitivity to noise and sampling [16]. At the scale factor τ, RCMSE considers τ different coarse-grained signals, corresponding to different starting points of the coarse-graining process [14]. Therefore, RCMSE yields more stable results in comparison with MSE. Nevertheless, both InMSE and RCMSE may lead to undefined values for short signals as a consequence of using SampEn in the second step of their algorithms [13]. Additionally, the SampEn-based approaches may not be fast enough for some real-time applications.

To deal with these deficiencies, multiscale DispEn (MDE) based on our introduced DispEn was developed [13]. Refined composite MDE (RCMDE) was then proposed to improve the stability of the MDE-based values [13]. It was found that MDE and RCMDE have the following advantages over MSE and RCMSE: (1) they are noticeably faster as a consequence of using DispEn with computational cost of O(*N*)—where *N* is the signal length—, compared with the O$\left(N^{2}\right)$ for SampEn; (2) they result in more stable profiles for synthetic and real signals; (3) MDE and RCMDE discriminate different kinds of physiological time series better than MSE and RCMSE; and (4) they do not yield undefined values [13].

**Figure 1:**
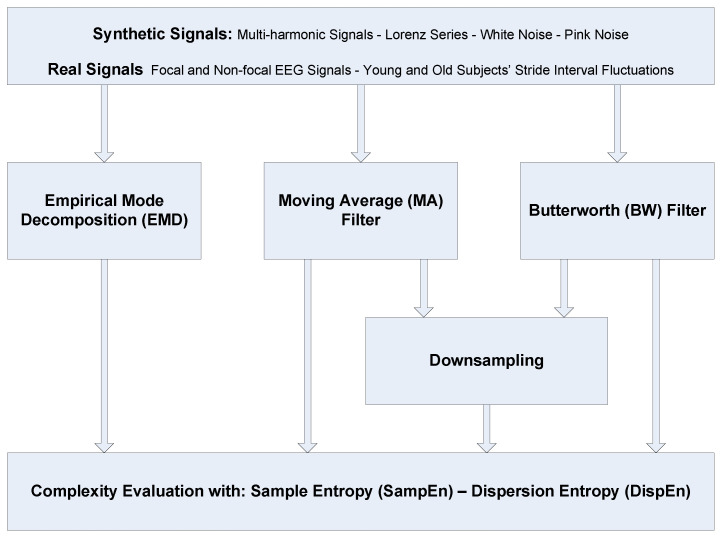
Flowchart of the alternatives to the coarse-graining method and the datasets used in this study.

The aim of this research is to contribute to the understanding of different alternatives to coarse-graining in complexity approaches. To this end, we first revise the frequency responses for the three main filtering processes (i.e., MA, BW, and EMD) used in such methods. The role of downsampling in the classical coarse-graining process, which has not been systematically explored yet, is then investigated in the article. We assess the impact of coarse-graining in multiscale entropy estimations based on both SampEn and DispEn. To compare these methods, several synthetic data and two real physiological datasets are employed. For the sake of clarity, a flowchart of the alternatives to the coarse-graining method in addition to the datasets used in this article is shown in Figure 1.

## 2. Multiscale Entropy-Based Approaches

The MSE- and MDE-based methods include two main steps: (1) coarse-graining process; and (2) calculation of SampEn and DispEn at each scale τ. For simplicity, we detail only the DispEn-based complexity algorithms. Likewise, the SampEn-based algorithms are defined.

### 2.1. MDE Based on Moving Average (MA) and Butterworth (BW) Filters with and without Downsampling (DS)

#### 2.1.1. Coarse-Graining Approaches

A coarse-graining technique with DS denotes a decimation by scale factor τ. Decimation is defined as two steps [17,18]: (1) reducing high-frequency time series components with a digital low-pass filter; and (2) DS the filtered time series by τ; that is, keep only one every τ sample points.

Assume that we have a univariate signal of length *L*: $u=\{u_{1},u_{2},\ldots ,u_{i},\ldots ,u_{L}\}$. In the coarse-graining process, the original signal **u** is first filtered by an MA—a low-pass finite-impulse response (FIR) filter—as follows:

(1) $${v_{\ell }}^{\left(\tau \right)}=\frac{1}{\tau }\sum_{k=0}^{\tau -1}u_{\ell +k},\;\;\;\;\;\;1\leq \ell \leq L-\tau +1.$$

The frequency response of the MA filter is as follows [19]:

(2) $$\left|H\left(e^{j2\pi f}\right)\right|=\frac{1}{\tau }\frac{sin(\pi f\tau )}{sin(\pi f)},$$

where *f* denotes the normalized frequency ranging from 0 to 0.5 cycles per sample (normalized Nyquist frequency). The frequency response of the MA filter has several shortcomings: (1) a slow roll-off of the main lobe; (2) large transition band; (3) and important side lobes in the stop-band. To alleviate these problems, a low-pass BW filter was proposed [11]. This filter provides a maximally flat (no ripples) response [19]. The squared magnitude of the frequency of BW filter is defined as follows:

(3) $${\left|H\left(e^{j2\pi f}\right)\right|}^{2}=\frac{1}{1+{\left(f/f_{c}\right)}^{2n}},$$

where $f_{c}$ and *n* denote the normalized cut-off frequency and filter order, respectively [11,19]. Herein, n=6 and $f_{c}=\frac{0.5}{\tau }$ [11]. The original signal **u** is filtered by BW filter. In fact, the low-pass filters eliminate the fast temporal scales (higher frequency components) to take into account progressively slower time scales (lower frequency components).

Next, the time series filtered by either MA or BW is downsampled by the scale factor τ. Assume the downsampled signal is $x^{\left(\tau \right)}=\left\{x_{j}^{\left(\tau \right)}\right\}\;\left(1\leq j\leq \left\lfloor \frac{L}{\tau }\right\rfloor =N\right)$.

In this study, we consider the coarse-graining process with and without DS. MSE and MDE with MA filter and without DS are respectively named MSE_MA_ and MDE_MA_. MSE_MA_ and MDE_MA_ with DS are termed MSE and MDE herein.

#### 2.1.2. Calculation of DispEn or SampEn at Every Scale Factor

The DispEn or SampEn value is calculated for each coarse-grained signal $x^{\left(\tau \right)}=\left\{x_{j}^{\left(\tau \right)}\right\}$. It is worth noting that MDE is more than the combination of the coarse-graining [3] with DispEn: the mapping based on the normal cumulative distribution function (NCDF) used in the calculation of DispEn [6] for the first temporal scale is maintained across all scales. That is, in MDE and RCMDE, μ and σ of NCDF are respectively set at the average and standard deviation (SD) of the original signal and they remain constant for all scale factors. This approach is similar to keeping the threshold *r* constant fixed (usually 0.15 of the SD of the original signal) in the MSE-based algorithms [3]. In a number of studies (e.g., [3,20]), it was found that keeping *r* constant is preferable to recalculating the threshold *r* at each scale factor separately.

### 2.2. Refined Composite Multiscale Dispersion Entropy (RCMDE)

At scale factor τ, RCMDE considers τ different coarse-grained signals, corresponding to different starting points of the coarse-graining process. Then, for each of these shifted series, the relative frequency of each dispersion pattern is calculated. Finally, the RCMDE value is defined as the Shannon entropy value of the averages of the rates of appearance of dispersion patterns of those shifted sequences [13]. The MA filter used in RCMDE and RCMSE may be substituted by the BW filter, respectively called RCMDE_BW_ and RCMSE_BW_ here.

### 2.3. Intrinsic Mode Dispersion Entropy (InMDE)

Due to the advantages of DispEn over SampEn for short signals, intrinsic mode DispEn (InMDE) based on the algorithm of InMSE is proposed herein. The algorithm of InMDE includes the following two key steps:

1. Calculation of the sum of the intrinsic mode functions (IMFs) obtained by EMD: In this step, the original signal **u** is decomposed to $IMF_{\alpha }\;\left(1\leq \alpha \leq \tau_{\max}-1\right)$ and a residual signal $IMF_{\tau_{\max}}=u-\sum_{\alpha =1}^{\tau_{\max}-1}IMF_{\alpha }$. It is worth noting that the first IMF, $IMF_{1}$, shows the highest frequency component in a signal, while the last IMF, $IMF_{\tau_{\max}}$, reflects the trend of the time series. Next, the cumulative sums of IMFs (CSI) for each scale factor τ are defined as follows [15]:
  (4) $${\mathrm{CSI}}^{\left(\tau \right)}\left(x\right)=\sum_{\lambda =\tau }^{\tau_{\max}}IMF_{\lambda },$$
  where $IMF_{\lambda }$ denotes the $\lambda^{th}$ IMF obtained by EMD. Thus, ${\mathrm{CSI}}^{\left(1\right)}$ is equal to the original signal **u**.
2. Calculation of DispEn of ${\mathrm{CSI}}^{\left(\tau \right)}\left(x\right)$ at each scale factor: The DispEn value is calculated at each scale factor. Like MDE and RCMDE, μ and σ of NCDF are respectively set at the average and SD of the original signal and they remain constant for all scale factors in InMDE.

It is worth noting that InMSE and InMDE do not downsample the filtered signals. That is, the number of samples for each ${\mathrm{CSI}}^{\left(\tau \right)}\left(x\right)$ is equal to that for the original signal, leading to more reliable results for higher scale factors. The complexity metrics for univariate signals and their characteristics are summarized in Table 1. The Matlab codes used in this study are described in Appendix.

### 2.4. Parameters of the Multiscale Entropy Approaches

For all the SampEn-based methods, we set d=1, m=2, and r=0.15 of the SD of the original signal [3]. For all the DispEn-based approaches, we set d=1 and c=6. For more information about *c* and *d*, please refer to [6,13].

For the DispEn-based complexity measures without DS, as the length of coarse-grained signals is equal to that of the original signal, it is advisable to follow $c^{m}<L$. For the SampEn-based complexity approaches without DS, it is recommended to have at least $10^{m}$ (or preferably $20^{m}$) sample points for the embedding dimension *m* [21,22].

For the DispEn-based multiscale approaches with DS, since the decimation process causes the length of a signal decreases to $\left\lfloor \frac{L}{\tau_{max}}\right\rfloor$, $c^{m}<\left\lfloor \frac{L}{\tau_{max}}\right\rfloor$ is recommended. Similarly, for the SampEn-based complexity techniques with DS, $10^{m}<\left\lfloor \frac{L}{\tau_{max}}\right\rfloor$ [3] is recommended.

On the other hand, in RCDME, we consider τ coarse-grained time series with length $\left\lfloor \frac{L}{\tau_{max}}\right\rfloor$. Therefore, the total sample points calculated in RCMDE is $\tau \times \left\lfloor \frac{L}{\tau_{max}}\right\rfloor \approx L$. Thus, RCMDE follows $c^{m}<L$, leading to more reliable results, especially for short signals. Likewise, it is advisable to have at least $10^{m}$ (or preferably $20^{m}$) sample points for RCMSE with embedding dimension *m*.

## 3. Evaluation Signals

In this section, the synthetic and real signals used in this study to evaluate the behaviour of the multiscale entropy approaches are described.

### 3.1. Synthetic Signals

White noise is more irregular than pink noise (1/f noise), although the latter is more complex because pink noise contains long-range correlations and its 1/f decay produces a fractal structure in time [3,5,8]. Therefore, white and pink noise are two important signals to evaluate the multiscale entropy techniques [3,5,8,23,24,25].

In order to investigate the change in the behavior of a nonlinear system, the Lorenz attractor is used. Further details can be found in [26,27]. To evaluate the effect of filtering and downsampling processes on different frequency components of time series, multi-harmonic signals are employed [16]. Finally, to inspect the effect of noise on multiscale approaches, white noise was added to the Lorenz and multi-harmonic time series.

### 3.2. Real Biomedical Datasets

Multiscale entropy techniques are broadly used to characterize physiological recordings [2,3,12,25]. To this end, electroencephalograms (EEGs) [28] and stride internal fluctuations [29] are used to distinguish different kinds of dynamics of time series.

#### 3.2.1. Dataset of Focal and Non-Focal Brain Activity

The ability of complexity measures to discriminate focal from non-focal signals is evaluated by the use of an EEG dataset (publicly-available at [30]) [28]. The dataset includes five patients and, for each patient, there are 750 focal and 750 non-focal bivariate time series. The length of each signal was 20 s with sampling frequency of 512 Hz (10,240 samples). For more information, please, refer to [28]. All subjects gave written informed consent that their signals from long-term EEG might be used for research purposes [28]. Before computing the entropies, the EEG signals were digitally band-pass filtered between 0.5 Hz and 150 Hz using a fourth-order Butterworth filter.

#### 3.2.2. Dataset of Stride Internal Fluctuations

To compare multiscale entropy methods, stride interval recordings are used [29,31]. The time series were recorded from five young, healthy men (23–29 years old) and five healthy old adults (71–77 years old). All the individuals walked continuously on level ground around an obstacle-free path for 15 min. The stride interval was measured by the use of ultra-thin, force sensitive resistors placed inside the shoe. For more information, please refer to [29].

## 4. Results and Discussion

### 4.1. Synthetic Signals

#### 4.1.1. Frequency Responses of Cumulative Sums of IMFs (CSI), and Moving Average (MA) and Butterworth (BW) Filters

**Figure 2:**
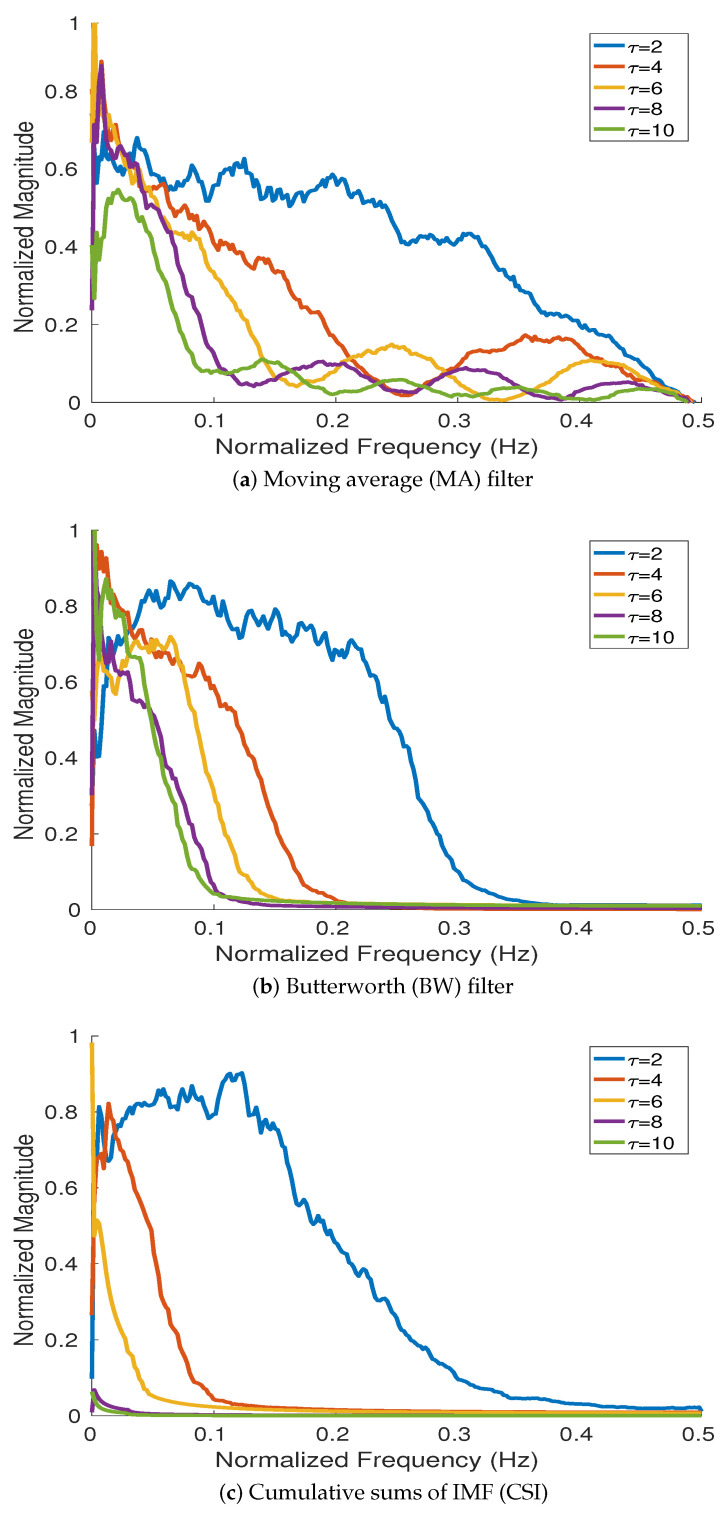
Magnitude of the frequency response for (**a**) MA, (**b**) BW, and (**c**) CSI at different scale factors (τ=2, 4, 6, 8, and 10) computed from 200 realizations of white noise with length 512 sample points.

To investigate the frequency responses of MA, BW, and CSI, we used 200 realizations of white noise with length 512 sample points following [32,33]. The average Fourier spectra obtained by MA, BW, and CSI at different scale factors (i.e., 2, 4, 6, 8, and 10) are depicted in Figure 2. The results show that BW, MA, and CSI can be considered as low-pass filters with different cut-off frequencies. The results for MA and BW filters are in agreement with their theoretical frequency responses shown in Equations (2) and (3), respectively. The results for CSI are also in agreement with the fact that $IMF_{1}$ corresponds to a half-band high-pass filter and $IMF_{\lambda }$ (λ≥2) can be considered as a filter bank of overlapping bandpass filters [33].

The magnitude of the frequency response for BW, compared with MA, is flatter in the passband, side lobes in its stopband are not present, and the roll-off is faster. Therefore, the filter’s frequency response leads to a more accurate elimination of the components with frequency above cut-off frequencies. This fact reduces aliasing while the filtered signals are downsampled. The behavior of the frequency response for CSI is similar to that for BW. However, the cut-off frequencies obtained by CSI are considerably smaller than those for BW. This fact results in very low entropy values at high scale factors.

#### 4.1.2. Effect of Different Low-Pass Filters on Multi-Harmonic and Lorenz Series

**Figure 3:**
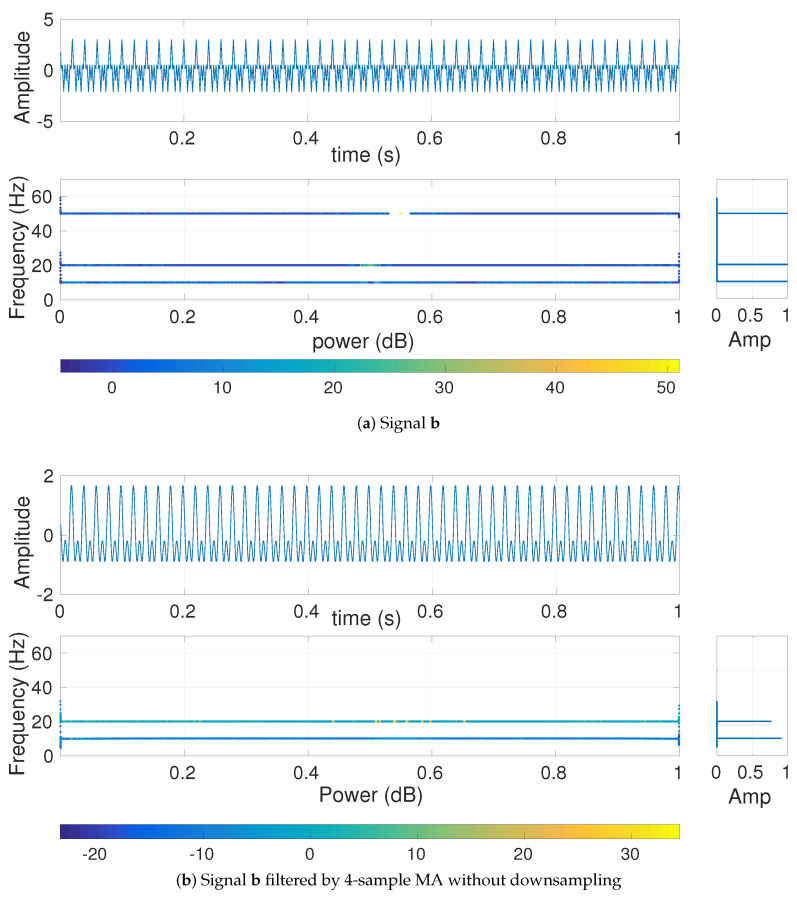
Hilbert transform of the decomposed VMD-based IMFs obtained from (**a**) $b_{i}=\cos\left(2\pi 10i\right)+\cos\left(2\pi 20i\right)+\cos\left(2\pi 50i\right)$ and (**b**) b filtered by 20-sample MA (scale 20).

To understand the effect of MA, BW, and CSI on multi-harmonic signals, we use $b_{i}=\cos\left(2\pi 10i\right)+\cos\left(2\pi 20i\right)+\cos\left(2\pi 50i\right)$ with sampling frequency 200 Hz and length 20 s. The first second of the signal **b** is depicted in Figure 3. To show the frequency components of b and their amplitude values, we used the combination of Hilbert transform and recently introduced variational mode decomposition (VMD). VMD is a generalization of the classic Wiener filter into adaptive, multiple bands [16]. After decomposing the original signals into its IMFs using VMD, we employ the Hilbert transform to find the instantaneous frequency of each IMF [16,34].

The frequency components of **b** and their corresponding amplitudes are depicted in Figure 3a. The Hilbert transform of b filtered by 4-sample MA (Figure 3b) illustrates that the harmonic cos(2π50i) is completely eliminated, in agreement with the fact that MA is a low-pass filter with cut-off frequency $\frac{f_{s}}{2\tau }$ and completely eliminates the frequency component $f_{z}$ at $\frac{f_{s}}{\tau }$ (here at $50=\frac{200}{4}$) based on Equation (2) [11].

**Figure 4:**
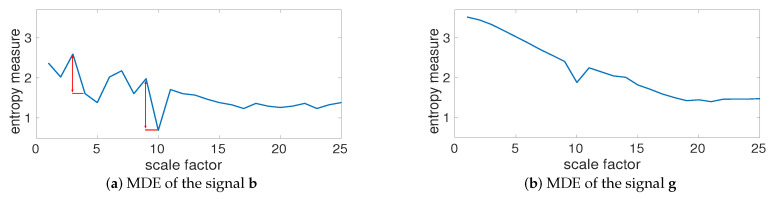
MDE values for (**a**) $b_{i}=\cos\left(2\pi 10i\right)+\cos\left(2\pi 20i\right)+\cos\left(2\pi 50i\right)$ and (**b**) $g_{i}=b_{i}+\eta$. The largest changes in entropy values (the red double arrows) occur at temporal scale 4 and 10 (respectively correspond to $50=\frac{200}{4}$ and $20=\frac{200}{10}$).

The MDE values for **b**, depicted in Figure 4a, show that the largest changes in entropy values occur at temporal scale 4 and 10 (based on $50=\frac{200}{4}$ and $20=\frac{200}{10}$—please see the red double arrows in Figure 4). In fact, the largest changes in entropy values are related to the main frequency components of a multi-harmonic time series. To investigate the effect of noise on MDE values, we created $g_{i}=b_{i}+\eta$, where η denotes a uniform random variable between 0 to 1. The MDE values for **g**, plotted in Figure 4b, illustrate a decrease at temporal scales from 1 to 19 and then the entropy values become approximately constant. This is in agreement with the fact that the smaller scale factors correspond to higher frequency components, whereas smaller scales correspond to lower frequencies [35]. Comparing Figure 4a,b shows that after filtering the effect of white noise by MA, the profiles for **b** and **g** are very close (temporal scales 19 to 25). This suggests that white noise affects lower temporal scales. It is worth noting that the behavior of MDE_BW_ and that of MDE_MA_ is similar.

**Figure 5:**
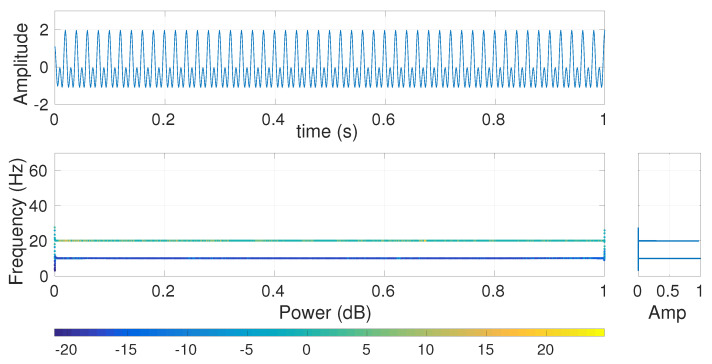
Hilbert transform of the decomposed VMD-based IMFs obtained from the signal **b** for CSI at scale 2.

However, the effect of CSI at scale 2 on **b** is shown in Figure 5. The results, compared with those for MA (see Figure 4b), illustrate similar behavior of CSI at scale 2 and MA at scale 4 in terms of the elimination of the highest frequency component of **b**. This is in agreement with the fact that, at a specific scale factor, the cut-off frequency for CSI is considerably lower than that for MA or BW (see Figure 2).

**Figure 6:**
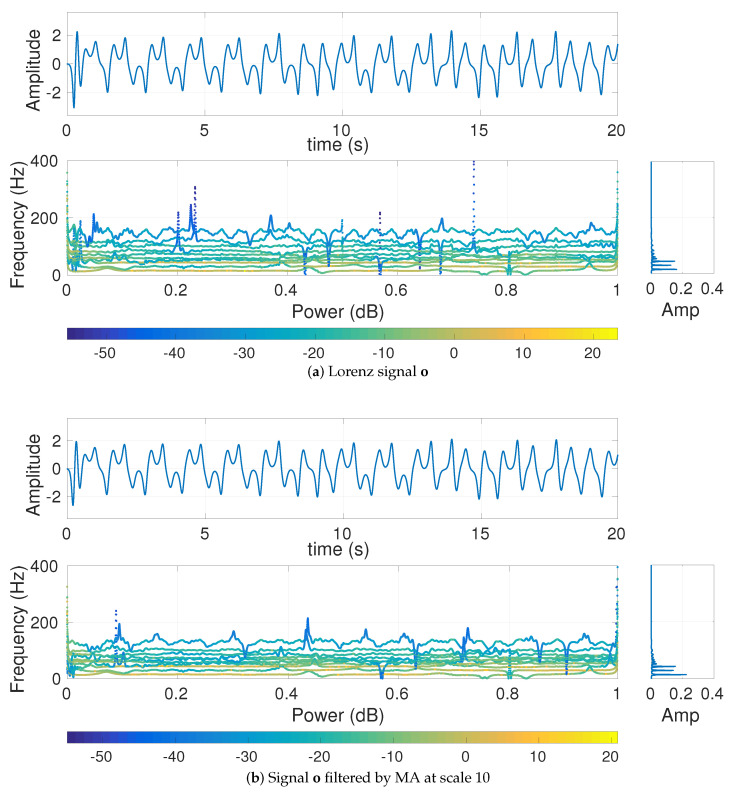
Hilbert transform of the decomposed VMD-based IMFs obtained from (**a**) the Lorenz signal o and (**b**) o filtered by MA at scale 10.

**Figure 7:**
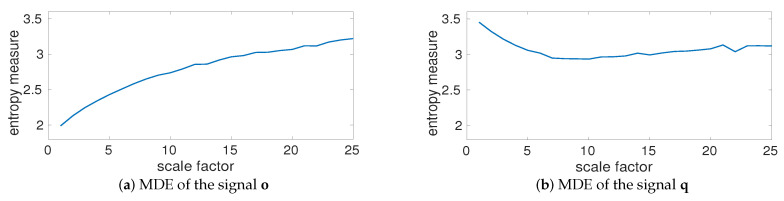
MDE results for (**a**) the Lorenz signal **o** and (**b**) $q_{i}=o_{i}+\eta$.

We also generated the Lorenz signal **o** with length 10,000 sample points and sampling frequency ($f_{s}$) 300 Hz. To have a nonlinear behavior, λ=10, $\beta =\frac{8}{3}$, and ρ=99.96 were set [26,27]. The signal **o** and o filtered by MA at scale 10 are shown in Figure 6. The MDE-based values for **o** are depicted in Figure 7a. The Nyquist frequency of the signal is ($\frac{300}{2}=150$) Hz and is close to its highest frequency component (around 150 Hz). Note that choosing a lower sampling frequency may result in aliasing. As the main frequency components of this time series are around 20–30 Hz, the MA filter is not able to completely eliminate the main frequency components of this signal at scale 10. It leads to the amplitude values of the filtered signal at scale 10 (without downsampling) being very close to those of the original time series **o**.

To inspect the effect of additive noise on MDE values, we created $q_{i}=o_{i}+\eta$, where η is a random variable between 0 to 1. The MDE values for **q**, plotted in Figure 7b, illustrate a decrease at low temporal scale and then an increase at high time scale factors. It is also found that the MDE values of **o** and **q** are approximately equal at scales between 18 to 25. This is also consistent with the fact that lower scale factors correspond to higher frequency components, whereas larger scales correspond to lower frequencies [35].

#### 4.1.3. Effect of Downsampling and Sampling Frequency on Multiscale Entropy Methods

**Figure 8:**
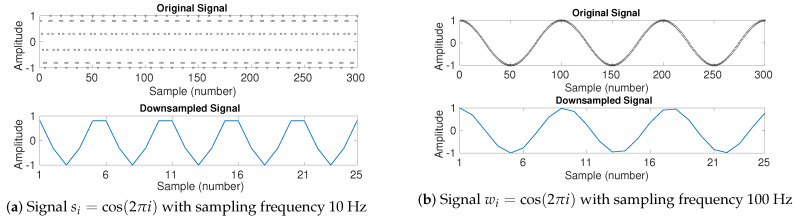
Downsampling the signal (**a**) $s_{i}=\cos\left(2\pi i\right)$ with length 300 sample points and sampling frequency 10 Hz, and (**b**) $w_{i}=\cos\left(2\pi i\right)$ with length 300 sample points and sampling frequency 100 Hz. The factor of downsampling is 12.

To investigate the effect of downsampling (without low-pass filtering) on multiscale entropy approaches, we created the signal $s_{i}=\cos\left(2\pi i\right)$ with length 300 sample points and sampling frequency 10 Hz, and (b) $w_{i}=\cos\left(2\pi i\right)$ with length 300 sample points and sampling frequency 100 Hz. The signals and their downsampled series by a factor of 12 are depicted in Figure 8.

When the sampling frequency of a time series is close to its main frequency components (see **s**—Figure 8a), the downsampled signal may have a lower frequency component in comparison with the original signal. It shows the effect of aliasing in the time series. Accordingly, the downsampled signals are more regular (have smaller entropy values). It is confirmed by the fact that the DispEn of **s** and its corresponding downsampled series are 2.0267 and 1.6058, respectively.

On the other hand, when the sampling frequency is high (see **w**—Figure 8b), the amplitude values of downsampled signal are approximately equal to those of the original signal. However, as the number of sample points decreases by 12, the rate of change along sample points is 12 times larger than that for the original signal. Thus, the original signal is more regular than its corresponding downsampled series. It is confirmed by the fact that the DispEn of **w** and its corresponding downsampled series are respectively 1.9618 and 2.5539.

#### 4.1.4. Multiscale Entropy Methods vs. Noise

**Figure 9:**
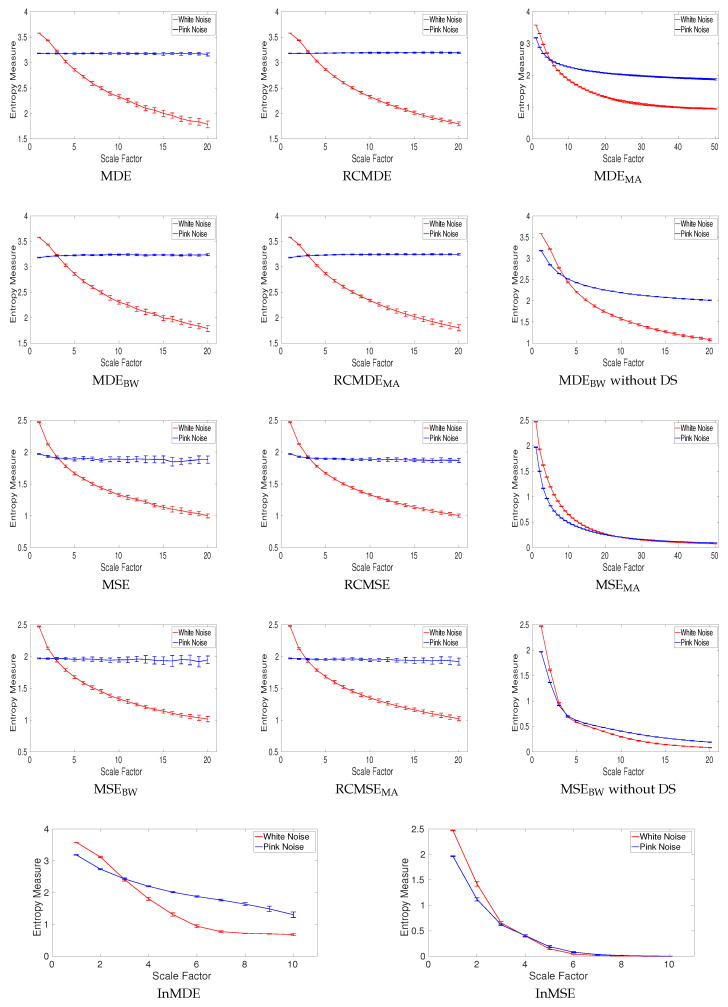
Mean value and SD of results obtained by the complexity measures computed from 40 different realizations of pink and white noise with length 8000 samples. Red and blue demonstrate white and pink noise, respectively.

**Figure 10:**
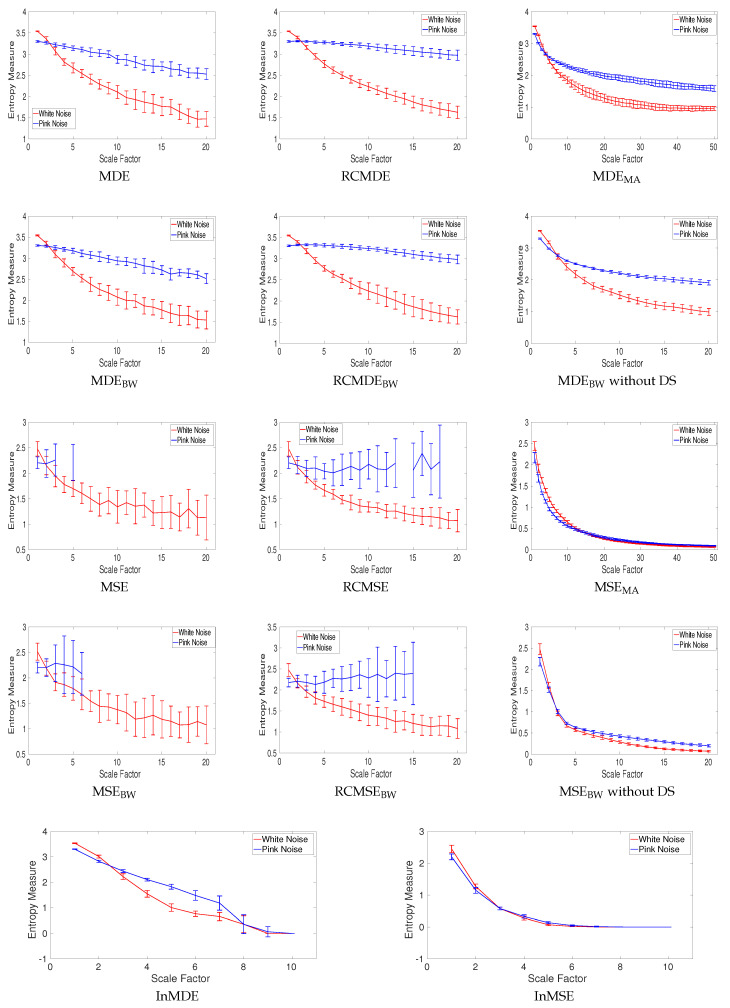
Mean value and SD of results obtained by the complexity measures computed from 40 different realizations of pink and white noise with length 400 samples. Entropy values obtained by MSE, RCMSE, MSE_BW_, and RCMSE_BW_ are undefined at several high scale factors. Red and blue demonstrate white and pink noise, respectively.

All of the complexity methods are used to distinguish the dynamics of white from pink noise. The mean and SD of results for the signals with length 8000 (long series) and 400 (short series) sample points are respectively depicted in Figure 9 and Figure 10. The results obtained by the complexity techniques with DS show that the entropy values decrease monotonically with scale factor τ for white noise. However, for pink noise, the entropy values become approximately constant over larger-scale factors. These are in agreement with the fact that, unlike white noise, 1/f noise has structure across temporal scale factors [3,5]. The profiles for MDE_MA_ and MSE_MA_ without DS, MDE_BW_ and MSE_BW_ without DS, InMSE, and InMDE decrease along the temporal scales as there is not a DS process to increase the rate of changes to increase entropy values. It should be mentioned that, as the crossing point of profiles for white and pink noise is at scale 23, $\tau_{\max}$ for the MA-based coarse graining is equal to 50. Furthermore, $\tau_{\max}$ for InMSE and InMDE is 10, as the entropy values at high scales are close to 0.

Entropy values obtained by MSE, RCMSE, MSE_BW_, and RCMSE_BW_ are undefined at high scale factors. Comparing Figure 9 and Figure 10 demonstrates that the longer the signals, the more robust the multiscale entropy estimations. The results also show that InMDE, compared with InMSE, better discriminates white from pink noise.

To compare the results obtained by the complexity algorithms, we used the coefficient of variation (CV) defined as the SD divided by the mean. We use such a metric as the SDs of signals may increase or decrease proportionally to the mean. The CV values at scale 10, as a trade-off between low and high scale factors, for noise signals with length 8000 and 400 sample points are respectively illustrated in Table 2 and Table 3. Of note is that we consider scale 25 and 5 for the MSE_MA_ and MDE_MA_, and InMSE and InMDE profiles, respectively. The refined composite technique decreases the CVs for all the MSE- and MDE-based algorithms, showing its advantage to improve the stability of results for short and long noise. The smallest CVs for long pink and white noise are our developed MDE_BW_ without DS and RCMDE_BW_ methods, respectively. The smallest CVs for short pink and white noise are achieved by RCMDE_BW_ and RCMDE, respectively. Overall, the smallest CVs are obtained by the DispEn-based complexity measures.

#### 4.1.5. Effect of Refined Composite on Nonlinear Systems without Noise

**Figure 11:**
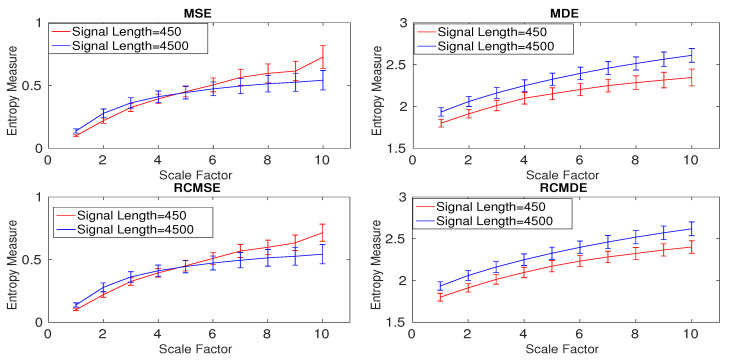
Mean and SD of the results obtained by the MSE, MDE, RCMSE, and RCMDE for the Lorenz series with lengths 450 and 4500 sample points.

To understand the effect of the refined composite technique on nonlinear signals without noise, we created 40 realizations of two Lorenz signals with lengths of 450 and 4500 sample points and sampling frequency ($f_{s}$) 150 Hz. To have a nonlinear behavior, the values of λ=10, $\beta =\frac{8}{3}$, and ρ=28 were used in the Lorenz system [26,27]. The results obtained by MSE, MDE, RCMSE, and RCMDE are depicted in Figure 11 and are in agreement with [25,27]. Of note is that the entropy values for RCMSE_BW_ and RCMDE_BW_ are similar to those for RCMSE and RCMDE, respectively. Thus, these results are not shown herein.

To investigate the effect of the refined composite technique on the stability of results, the CVs for the multiscale approaches at scale 5 are calculated. The smallest CVs, illustrated in Table 4 are obtained by MDE and RCMDE approaches. The results also suggest that the refined composite does not improve the stability of profiles for the signal with length 4500 samples (long signals). For the Lorenz series with length 450 sample points, RCMSE and RCMDE lead to smaller CV values in comparison with MSE and MDE, in that order, showing the importance of the refined composite method to characterize small time series.

### 4.2. Real Signals

#### 4.2.1. Dataset of Focal and Non-Focal Brain Activity

**Figure 12:**
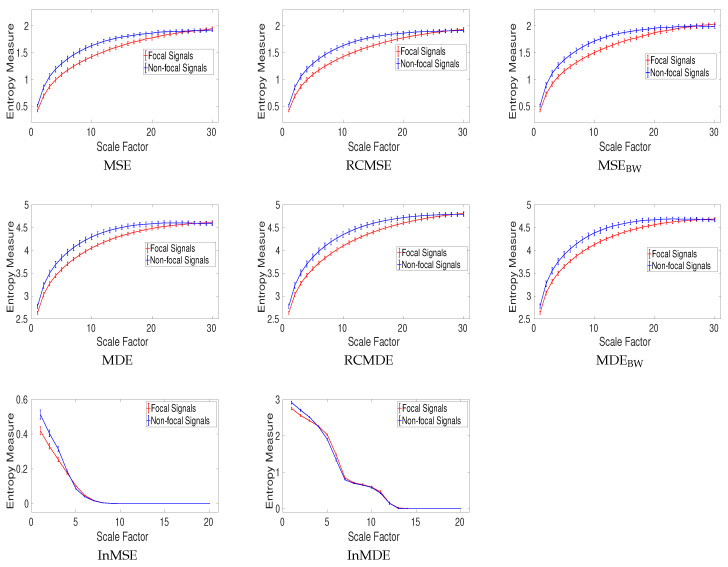
Mean value and SD of results obtained by the MSE, MDE, RCMSE, RCMDE, MSE_BW_, MDE_BW_, InMSE, and InMDE computed from the focal and non-focal EEGs.

For the focal and non-focal EEG dataset, the results obtained by MSE, MDE, RCMSE, RCMDE, MSE_BW_, MDE_BW_, InMSE, and InMDE, depicted in Figure 12, show that the non-focal signals are more complex than the focal ones. This fact is in agreement with previous studies [28,36].

The results for RCMSE_BW_ and RCMDE_BW_ were respectively similar to those for MSE_BW_ and MDE_BW_. Thus, they are not shown herein. Note that, for MDE and RCMDE, $\tau_{max}$ and *m*, respectively, were 30 and 3. It should also be mentioned that the average entropy values over two channels for these bivariate EEG signals are reported for the univariate complexity techniques.

To compare the results, the CV values obtained by the univariate multiscale approaches, except InMSE and InMDE, are calculated at scale factor 15. These are shown in Table 5. The CV values for MDE, RCMDE, MSE, and RCMSE illustrate that the refined composite approach does not enhance the stability of the MDE and MSE profiles. Overall, the smallest CV values are achieved by DispEn-based complexity methods.

#### 4.2.2. Dataset of Stride Internal Fluctuations

**Figure 13:**
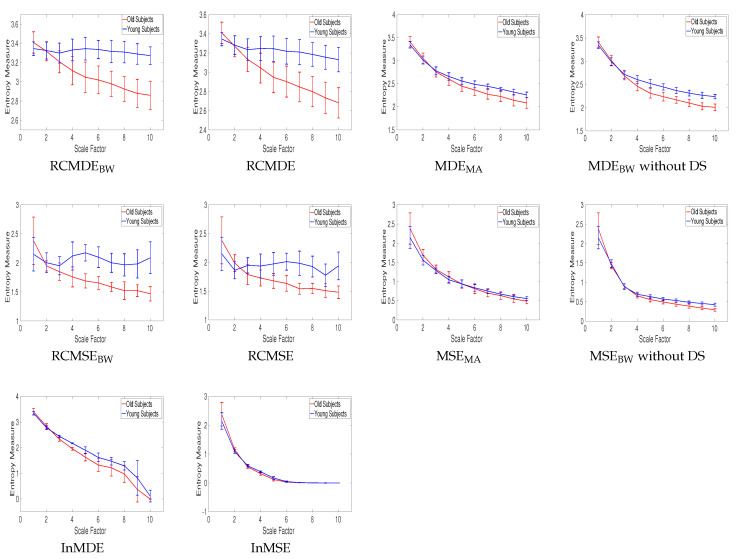
Mean value and SD of results obtained by the complexity measures computed from the young and old subjects’ stride interval recordings.

In Figure 13, the mean and SD of the RCMDE_BW_, RCMDE, MDE_MA_, MDE_BW_ without DS, InMDE, RCMSE_BW_, RCMSE, MSE_MA_, MSE_BW_ without DS, and InMSE values computed from young and old subjects’ stride internal fluctuations are illustrated. As the number of samples for these time series are between 400 to 800 sample points, we do not use MSE, MDE, MSE_BW_, and MDE_BW_.

For each scale factor, the average of entropy values for elderly subjects is smaller than that for young ones, in agreement with those obtained by the other entropy-based methods [37] and the fact that recordings from healthy young subjects correspond to more complex states because of their ability to adapt to adverse conditions, whereas aged individuals’ signals present complexity loss [3,5,38]. The results also suggest that, when dealing with short signals, the complexity measures without downsampling (i.e., MSE_MA_, MDE_MA_, and MSE_BW_ and MDE_BW_ without DS) are appropriate to distinguish different kinds of dynamics of real signals.

The CV values at those scales whose profiles do not have an overlap are illustrated in Table 6. It is found that MDE_BW_ without DS leads to the smallest CV values.

## 5. Time Delay, Downsampling, and Nyquist Frequency

According the previous complexity-based approaches [2,3,13,15], the time delay was equal to 1 in this study. Nevertheless, if the sampling frequency is considerably larger than the highest frequency component of a signal, the first minimum or zero crossing of the autocorrelation function or mutual information can be used for the selection of an appropriate time delay [39].

Alternatively, a signal may be downsampled before calculating the complexity-based entropy approaches to adjust its highest frequency component to its Nyquist frequency ($f_{s}/2$) [40]. Accordingly, when the coarse-graining process starts, the low-pass filtering will affect the highest frequency component of the signal at low temporal scale factors. It is worth noting that if the main frequency components of the signal are considerably lower than its highest frequency component (e.g., the signal **o** - please see Figure 7), the filtering process may make only a little change in the amplitude values of the signal at even large scales.

## 6. Future Work

Wavelet transform, which is a powerful filter bank broadly used for analysis of non-stationary recordings, can be employed to decompose a signal to several series with specific frequency bands [41]. Accordingly, the wavelet-based filter bank could be used as a complexity approach. VMD can also be used as an alternative to EMD in InMSE and InMDE. VMD, unlike EMD, provides a solution to the decomposition problem that is theoretically well founded and more robust to noise than EMD [16]. A recent development in the field has tried to generalize multivariate and univariate multiscale algorithms to a family of statistics by using different moments (e.g., variance, skewness, and kurtosis) in the univariate and multivariate coarse-graining process [25,42,43,44]. It is recommended to compare these techniques in the context of signal processing and to investigate their interpretations. As the existing univariate and even multivariate coarse-graining processes filter only series in each channel separately [38,43,45], there is a need to propose new multivariate filters dealing with the spatial and time domains at the same time.

## 7. Conclusions

In summary, we have compared existing and newly proposed coarse-graining approaches for univariate multiscale entropy estimation. Our results indicate that, as expected due to the filter bank properties of the EMD [33] in comparison with moving average and Butterworth filtering, the cut-off frequencies at each temporal scale τ of the former are considerably smaller than those for the latter. Therefore, InMSE and our developed InMDE have entropy values very close to 0 for relatively low values of temporal scales due to the exponential, rather than linear, dependency of the bandwidth at each scale. We also inspected the effect of the downsampling in the coarse-graining process in the entropy values, showing that it may lead to increased or decreased values of entropy depending on the sampling frequency of the time series.

Our results confirmed previous reports indicating that, when dealing with short or noisy signals, the refined composite approach [14,25] may improve the stability of entropy results. On the other hand, for long signals with relatively low levels of noise, the refined composite method makes little difference in the quality of the entropy estimation at the expense of a considerable additional computational cost. In any case, the use of dispersion entropy over sample entropy in the estimations led to more stable results based on CV values and ensured that the entropy values were defined at all temporal scales.

Finally, the profiles obtained by the multiscale techniques with and without downsampling led to similar findings (e.g., pink noise is more complex than white noise based on all the complexity methods) although the specific values of entropy may differ depending on the coarse-graining used. This suggests that downsampling within the coarse-graining procedure may not be needed to quantify the complexity of signals, especially for short ones. In fact, these kinds of techniques still eliminate the fast temporal scales to deal with progressively slower time scales as τ increases and take into account multiple time scales inherent in time series.

On the whole, it is expected that these findings contribute to the ongoing discussion regarding the development of stable, fast, and less sensitive-to-noise complexity approaches appropriate for either short or long time series. We recommend that future studies explicitly justify their choices for coarse-graining procedure in the light of the characteristics of the signals under analysis and the hypothesis of the study, and that they discuss their findings on the light of the behaviour of the selected entropy metric and coarse-graining procedure. 

## Figures and Tables

**Table 1:** Characteristics of the complexity metrics for univariate signals.

Methods	Filtering	Downsampling	Applicability of Refined Composite
MSE [2] and MDE [13]	Moving average	yes	yes
MSE_MA_ and MDE_MA_	Moving average	no	no
MSE_BW_ [11] and MDE_BW_	Butterworth	yes	yes
MSE_BW_ [11] and MDE_BW_ without downsampling	Butterworth	no	no
InMSE [15] and InMDE	Cumulative sums of IMFs	no	no

**Table 2:** CV values obtained by the complexity measures at scale factor 10 for forty realizations of pink and white noise with length 8000 sample points. Note that the scales 25 and 5 are considered for MSE_MA_ and MDE_MA_, and InMSE and InMDE, respectively.

**Noise**	**MDE**	**RCMDE**	**MDE_MA_ (Scale 25)**	**MDE_BW_**	**RCMDE_BW_**	**MDE_BW_ without DS**	**InMDE (Scale 5)**
Pink	0.0058	0.0038	0.0069	0.0044	0.0038	0.0031	0.0091
White	0.0174	0.0124	0.0246	0.0166	0.0115	0.0182	0.0394
**Noise**	**MSE**	**RCMSE**	**MSE_MA_ (Scale 25)**	**MSE_BW_**	**RCMSE_BW_**	**MSE_BW_ without DS**	**InMSE (Scale 5)**
Pink	0.0186	0.0105	0.0131	0.0176	0.0124	0.0130	0.0982
White	0.0201	0.0133	0.0135	0.0219	0.0203	0.0308	0.1330

**Table 3:** CV values obtained by the complexity measures at scale factor 10 for forty realizations of pink and white noise with length 400 sample points. Note that the scales 25 and 5 are considered for MSE_MA_ and MDE_MA_, and InMSE and InMDE, respectively.

**Noise**	**MDE**	**RCMDE**	**MDE_MA_ (Scale 25)**	**MDE_BW_**	**RCMDE_BW_**	**MDE_BW_ without DS**	**InMDE (Scale 5)**
Pink	0.0317	0.0194	0.0473	0.0320	0.0141	0.0204	0.0522
White	0.0726	0.0415	0.1116	0.0929	0.0876	0.0726	0.1435
**Noise**	**MSE**	**RCMSE**	**MSE_MA_ (Scale 25)**	**MSE_BW_**	**RCMSE_BW_**	**MSE_BW_ without DS**	**InMSE (Scale 5)**
Pink	undefined	0.1327	0.0434	undefined	0.2008	0.0822	0.2351
White	0.2385	0.0738	0.0605	0.2024	0.1736	0.1060	0.3779

**Table 4:** CVs of MSE, RCMSE, MDE, and RCMDE values for the 40 different realizations of the Lorenz signals with length 450 and 4500 samples at scale five.

Signal Length	MSE	MDE	RCMSE	RCMDE
450 sample points	0.1000	0.0898	0.0700	0.0309
4500 sample points	0.1156	0.0310	0.1134	0.0312

**Table 5:** CVs of MSE, RCMSE, MSE_BW_, MDE, RCMDE, and MDE_BW_ values for the focal and non-focal EEGs at scale 15.

Signals	MSE	RCMSE	MSE_BW_	MDE	RCMDE	MDE_BW_
Focal EEGs	0.0229	0.0229	0.0224	0.0083	0.0089	0.0083
Non-focal EEGs	0.0178	0.0191	0.0172	0.0111	0.0121	0.0109

**Table 6:** CV values obtained by the complexity measures for the stride interval recordings for young and old subjects.

Signals	RCMDE_BW_	RCMDE	MDE_BW_ without DS	RCMSE_BW_
Young subjects	0.0355	0.0410	0.0334	0.0644
Old subjects	0.0517	0.0540	0.0449	0.0723

## Appendix. Matlab Codes used in this Article

The Matlab codes of DispEn and MDE are available at https://datashare.is.ed.ac.uk/handle/10283/2637. The codes of SampEn and MSE can be found at https://physionet.org/physiotools/matlab/wfdb-app-matlab/. The code of EMD is also available at http://perso.ens-lyon.fr/patrick.flandrin/emd.html. For the Butterworth filter, we used the functions “butter” and “filter” in Matlab R2015a.
